# Belemnites of the family Belemnitellidae Pavlow, 1914 from the Late Cretaceous Maastrichtian stage in the Northern Hemisphere

**DOI:** 10.1017/njg.2024.15

**Published:** 2024-09-18

**Authors:** Norbert Keutgen, Zbyszek Remin

**Affiliations:** 1 Institute of Vegetables and Ornamentals, Department of Crop Sciences, University of Natural Resources and Life Sciences, Vienna, Austria; 2 Faculty of Geology, University of Warsaw, Warsaw, Poland

**Keywords:** Maastrichtian, *Belemnitella*, *Belemnella*, *Neobelemnella*, *Fusiteuthis*

## Abstract

The currently defined Global Stratotype Section and Point for the Campanian/Maastrichtian boundary at Tercis (France) lacks any belemnite record. However, the detailed correlation of Tercis with the Kronsmoor section in northern Germany has enabled recognising this boundary in terms of belemnite stratigraphy close to the first appearance datum (FAD) of *Belemnella obtusa* Schulz, 1979. Originally, the FAD of the genus *Belemnella* Nowak, 1913 (e.g. of *Belemnella lanceolata* (von Schlotheim, 1813)) has been widely used for defining the base of the traditionally understood Maastrichtian stage in the Boreal Realm. *Belemnella* appeared almost contemporaneously across a significant portion of epicontinental Europe in what is now considered topmost Campanian and dominated the lower Maastrichtian belemnite assemblages, co-occurring with *Belemnitella* d’Orbigny, 1840 and rare *Fusiteuthis* Kongiel, 1962. It disappeared in Western and Central Europe during the mid-Maastrichtian, and as a consequence, the FAD of *Belemnitella junior* Nowak, 1913 served as a biomarker defining the base of the upper Maastrichtian substage. It is only at the end of the Maastrichtian that the genus *Neobelemnella* Naidin, 1975 became abundant, replacing *Belemnitella* steadily from the east to the west as the dominating species. The factors underlying those remarkable shifts in belemnite assemblages remain uncertain, but various environmental elements, especially water depth and temperature, in addition ocean currents, and oceanic chemical composition are considered influential.

## Introduction

The first appearance datum (FAD) of the genus *Belemnella* Nowak, 1913 − specifically *Belemnella lanceolata* (von Schlotheim, 1813) − has traditionally been used to define the base of the Maastrichtian stage in the Boreal Realm in Central and Western Europe. In most areas of Europe, *Belemnella* almost completely replaced the genus *Belemnitella* d’Orbigny, 1840 during the latest Campanian, but in areas like the Middle Vistula River region (Poland) and the Maastricht region (the Netherlands), *Belemnella* coexisted with *Belemnitella* during the Early Maastrichtian (Kongiel, 1962; Keutgen & van der Tuuk, 1991; Remin, 2012, 2015). The disappearance of *Belemnella* during the mid-Maastrichtian is not well understood and − because considerable progress has been made in the correlation of sections especially in Western and Central Europe applying δ^13^C stable isotope stratigraphy (e.g. Niebuhr et al., 2011; Thibault et al., 2012; Voigt et al., 2012; Linnert et al., 2019; Wilmsen et al., 2019; Vellekoop et al., 2022) − there is growing evidence that it did not happen contemporaneously everywhere (see Discussion). It is plausible that a combination of environmental factors, such as temperature changes, sea-level fluctuations and/or modifications of ocean currents, played a significant role (Remin, 2018; Remin et al., 2022a, 2022b). During the late Maastrichtian, representatives of the genus *Neobelemnella* Naidin, 1975 successively expanded their area of distribution from east to west, displacing representatives of the genus *Belemnitella*. In order to complicate the situation further, there are currently two competing interpretations for the origin of *Neobelemnella*, namely that it evolved either locally from a Russian or Central Asian species of the genus *Belemnella* or represents a descendent from *Belemnitella*/?*Neobelemnella subfusiformis* a latest Campanian to earliest Maastrichtian species from New Jersey, western USA (Keutgen & Keutgen, 2020).

As a matter of fact, belemnite research is in a state of flux these days because advances involving AI-technology have fundamentally questioned earlier systematic approaches. Remin (2012, 2015) developed an innovative concept of belemnite determination and overcame the problem of the *a priori* subdivision of belemnite guards into species before statistical treatments (Christensen, 1975, 1995; Schulz, 1979). The artificial neural networks approach, the self-organising Kohonen algorithm creates groups of similar specimens (based on measurable features = similar input data) that may be regarded as so-called morphogroups, which may be interpreted as being composed of representatives of a paleontological/biological species. Remin developed a concept that allows determining guards of *Belemitella, Belemnella, Neobelemnella* and, possibly, also *Fusiteuthis* Kongiel, 1962 applying the same approach. For the genus *Belemnella*, it turned out that the methods of Remin (2012) and Schulz (1979) resulted in different species concepts (Schulz, 1979; Niebuhr et al., 2011; Remin, 2012; Keutgen et al., 2012). Even more significant, Remin’s (2018) study of the belemnites from the upper lower Maastrichtian of Hrebenne (southeast Poland) revealed the presence of two species, *Belemnella praearkhangelskii* Naidin, 1964a and *Belemnella kajnarensis* Naidin, 1964a, which cannot be separated applying the systematic approach of Schulz (1979). Both results substantially question determinations of *Belemnella* in Western and Central Europe based on the system of Schulz (1979). In addition, the determinations of eastern European belemnite species in studies such Naidin (1952, 1964a, 1974) and Baraboshkin et al. (2021, 2024) are difficult to assess because the basis of their systematic approach is not clear, usually not representing a population statistical concept at all.

It follows that for a thorough review of *Belemnella*, only the fauna of the Middle Vistula River valley is currently available, supplemented by sections in the immediate vicinity. The composed Middle Vistula valley section is an ideal reference due to its central location between Eastern and Western Europe because influences from both regions can be expected having an impact on the Middle Vistula belemnite fauna.

In order to compare the first and last appearance datum (LAD) of belemnites in different regions, carbon isotope data are used for correlation purpose when available (e.g. Niebuhr et al., 2011; Thibault et al., 2012; Voigt et al., 2012; Linnert et al., 2019; Wilmsen et al., 2019), otherwise calcareous nannofossils are used. In the Kronsmoor quarry ‘Saturn’, the base of the Maastrichtian corresponds to the marl layer mb609, a horizon close to the base of the *Belemnella obtusa* Zone, 12.5 m above flint layer F600 (Wilmsen et al., 2019). Exactly at the same stratigraphic level the boundary was placed in the Middle Vistula River Valley section, Poland (Remin, 2012). The potential lower-upper Maastrichtian substage boundary for years was defined by the FAD of *Belemnitella junior* Nowak, 1913 − however, the boundary has not been formally defined yet. For the convenience of this review, and for placing the belemnite record in an independent framework, reference is made to the position of the lower-upper Maastrichtian boundary as identified by Boussaha et al. (2016) in the Danish Chalk with the FAD of the calcareous nannofossil *Lithraphidites quadratus* Bramlette & Martini, 1964, dated at that locality at *c*. 68.8 Myr, although the position of the FAD of *L. quadratus* is globally highly diachronous (Thibault et al., 2012, fig. 8; Boussaha et al., 2016, fig. 10). When δ^13^C stable isotope data are available, a correlation of the lower-upper Maastrichtian boundary level as defined in the Stevns-1 borehole is here considered (Voigt et al., 2012; Vellekoop et al., 2022; Dubicka et al., 2023), in all other cases the FAD of *L. quadratus* is taken as an approximation of this boundary. In addition, upper Maastrichtian belemnite records in the Boreal Realm are linked to the FADs of *Nephrolithus frequens* Górka, 1957 and *Cribrosphaerella daniae* Perch-Nielsen, 1973 (Thibault, 2016, fig. 6).

### *Genus* Belemnella Nowak, 1913

**Figure 1:**
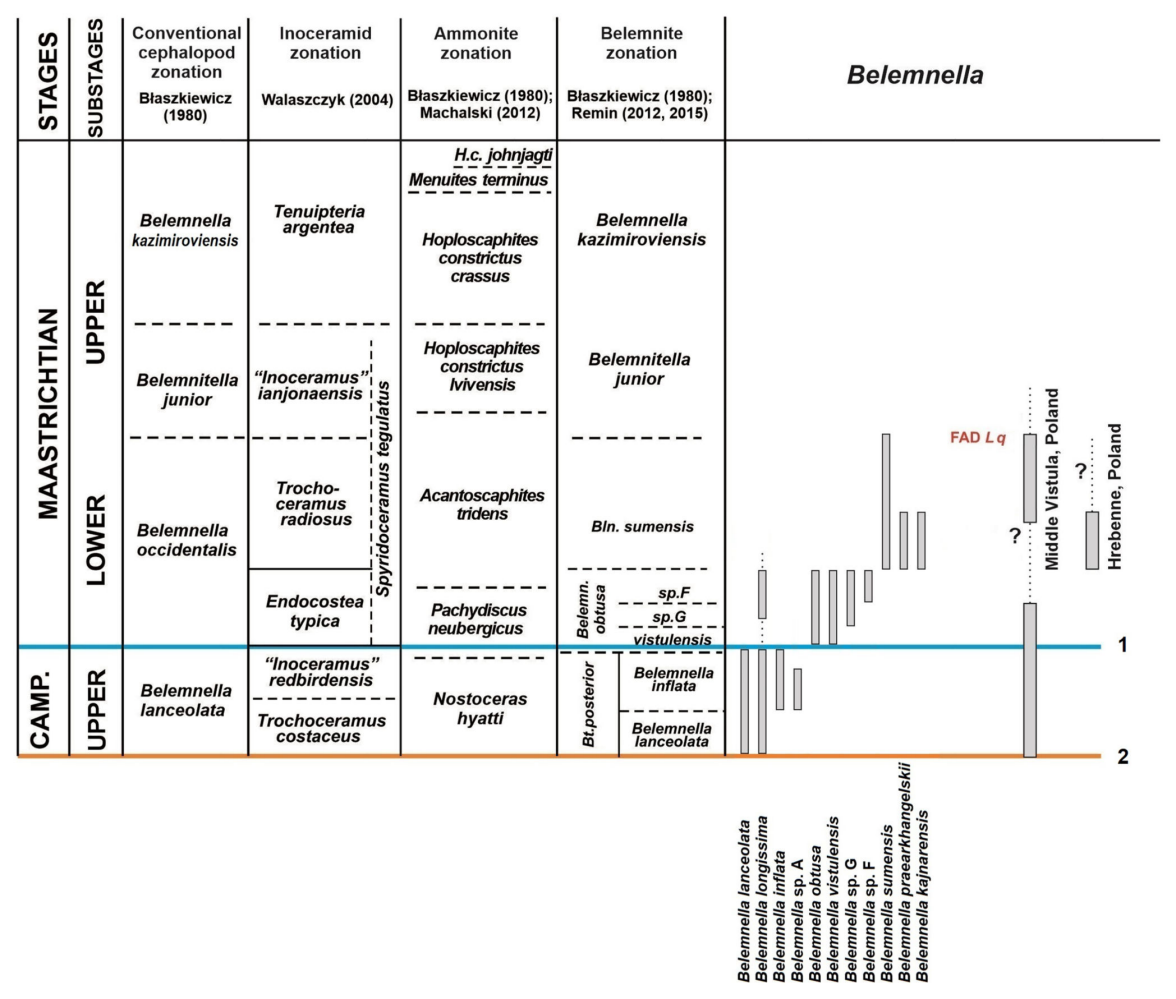
Stratigraphic ranges of *Belemnella* species and proposed belemnite zonation for the Middle Vistula valley section, central Poland; base of the Maastrichtian Stage according to the GSSP at Tercis, France (1) and as conventionally based on belemnites for the Boreal Realm (2). (L q: *Lithraphidites quadratus*).

**Figure 2:**
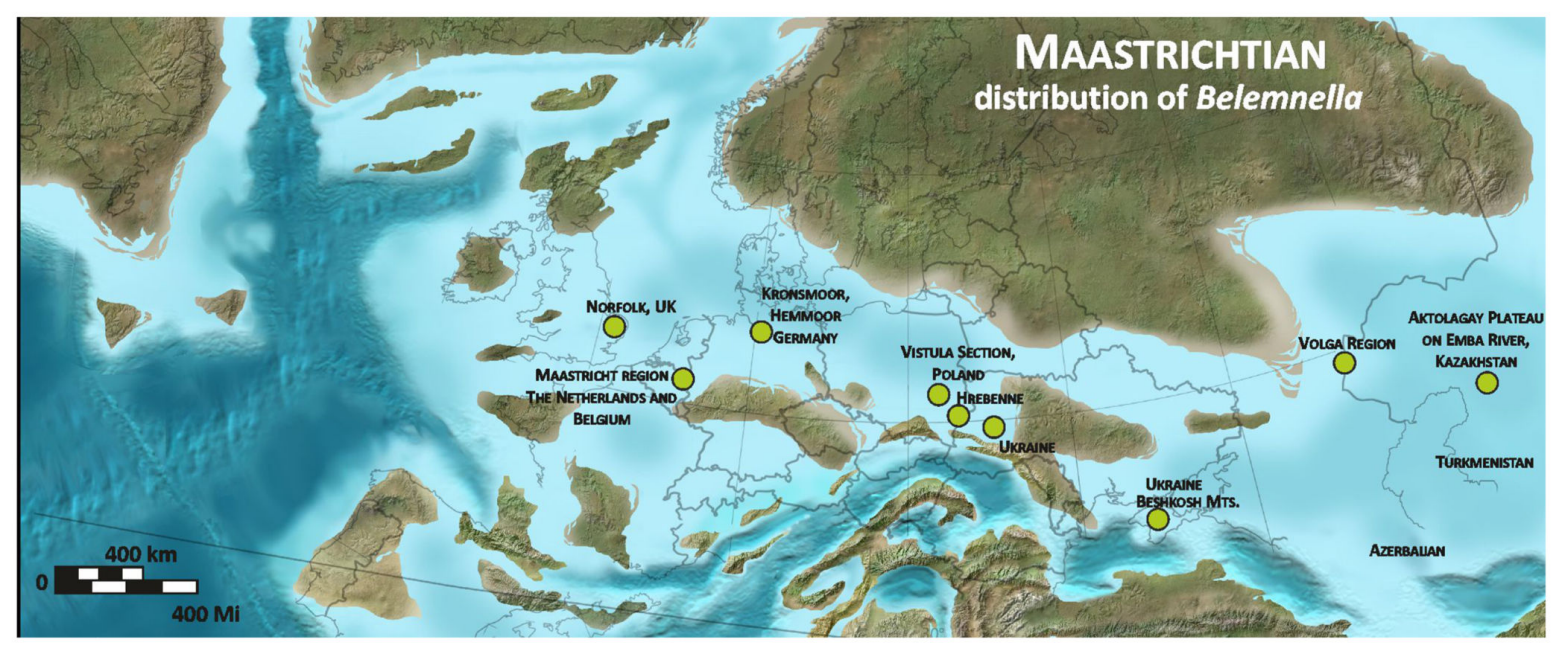
Paleogeographic distribution of the representatives of *Belemnella*. Used with permission of Colorado Plateau Geosystems Inc.Global Paleogeography and Tectonics in Deep Time © 2016.

The currently accepted official definition for the base of the Maastrichtian stage was published by Odin & Lamaurelle (2001) for the Global Stratotype Section and Point (GSSP) of the Campanian-Maastrichtian boundary at Tercis (France) and correlated with the marl layer mb609 in the Kronsmoor quarry ‘Saturn’ in northern Germany, close to the base of the *Belemnella obtusa* Zone, 12.5 m above flint layer F600 (Wilmsen et al., 2019). This position is distinctly above the classical definition of the boundary by the appearance of *Belemnella* at the flint layer F600 (Fig. 1). From the oldest *Belemnella* Zone, the uppermost Campanian *Belemnella lanceolata* Zone, only two species are known, *Belemnella lanceolata* (von Schlotheim, 1813) and *Belemnella longissima* Schulz, 1979, the earliest representatives of the latter species being known only from Kronsmoor and probably Balsvik, Sweden (Remin, 2012). The fact that already two species are reported from the stratigraphic oldest *Belemnella* Zone indicates that adaptive radiation in *Belemnella* began early in the latest Campanian and that local species also developed in other areas, which are not discussed here (Fig. 2). For the sake of completeness, however, the Russian *Belemnella licharewi* Jeletzky, 1941 should be mentioned because it is often cited as one of the stratigraphically oldest *Belemnella* species on the Russian Platform (Christensen, 1997a, 1997b).

From the stratigraphical youngest Campanian *Belemnella inflata* Zone already four species of *Belemnella* are known from the Middle Vistula section (five from Kronsmoor), of which only one species (*Bl. longissima*) was able to pass the Campanian-Maastrichtian boundary. Yet, four new species appeared successively within the lowermost Maastrichtian *Belemnella obtusa* Zone (Fig. 1). This reflects either another period of rapid adaptive radiation or alternatively migration of species from other areas into the Middle Vistula valley.

The *Belemnella* fauna as exposed at Hrebenne may be regarded typical of the ‘mid’ lower Maastrichtian (Fig. 1). Three species have been identified: *Belemnella sumensis* Jeletzky, 1949, *Bl. praearkhangelskii* and *Bl. kajnarensis* (Remin, 2018). The index species of the *Belemnella sumensis* Zone has also been recorded from Boiska in the Middle Vistula Valley (Kongiel, 1962 (as *Bl. occidentalis*), and unpublished specimens are housed in the coll. Remin). At Boiska, stratigraphically younger strata than at the Hrebenne section are exposed. Because the FAD of the calcareous nannofossil *L. quadratus* is also known from Boiska, a position close to the lower-upper Maastrichtian boundary can be deduced (see also Peryt et al., 2022; Dubicka et al., 2023). Noteworthy, *Bl. sumensis* is the only species of *Belemnella* in the uppermost Lower Maastrichtian of the Middle Vistula River section, potentially indicating a decrease in local species diversity of *Belemnella* before its extinction close to the top of the Lower Maastrichtian, at least in Western and Central Europe.

In summary, four *Belemnella* zones can be identified in the uppermost Campanian and lower Maastrichtian of the Middle Vistula valley. The upper Campanian *lanceolata* and *inflata* zones and the Lower Maastrichtian *obtusa* and *sumensis* zones. The duration of these zones may be roughly estimated from the numerical ages deduced for northern Germany (Voigt & Schönfeld, 2010; Thibault et al., 2012): *lanceolata* Zone *c*. 200 kyr; *inflata* Zone *c*. 600 kyr; *obtusa* Zone *c*. 1.4 Myr; *sumensis* Zone *c*. 2.4 Myr.

### *Genus* Belemnitella d’Orbigny, 1840

**Figure 3:**
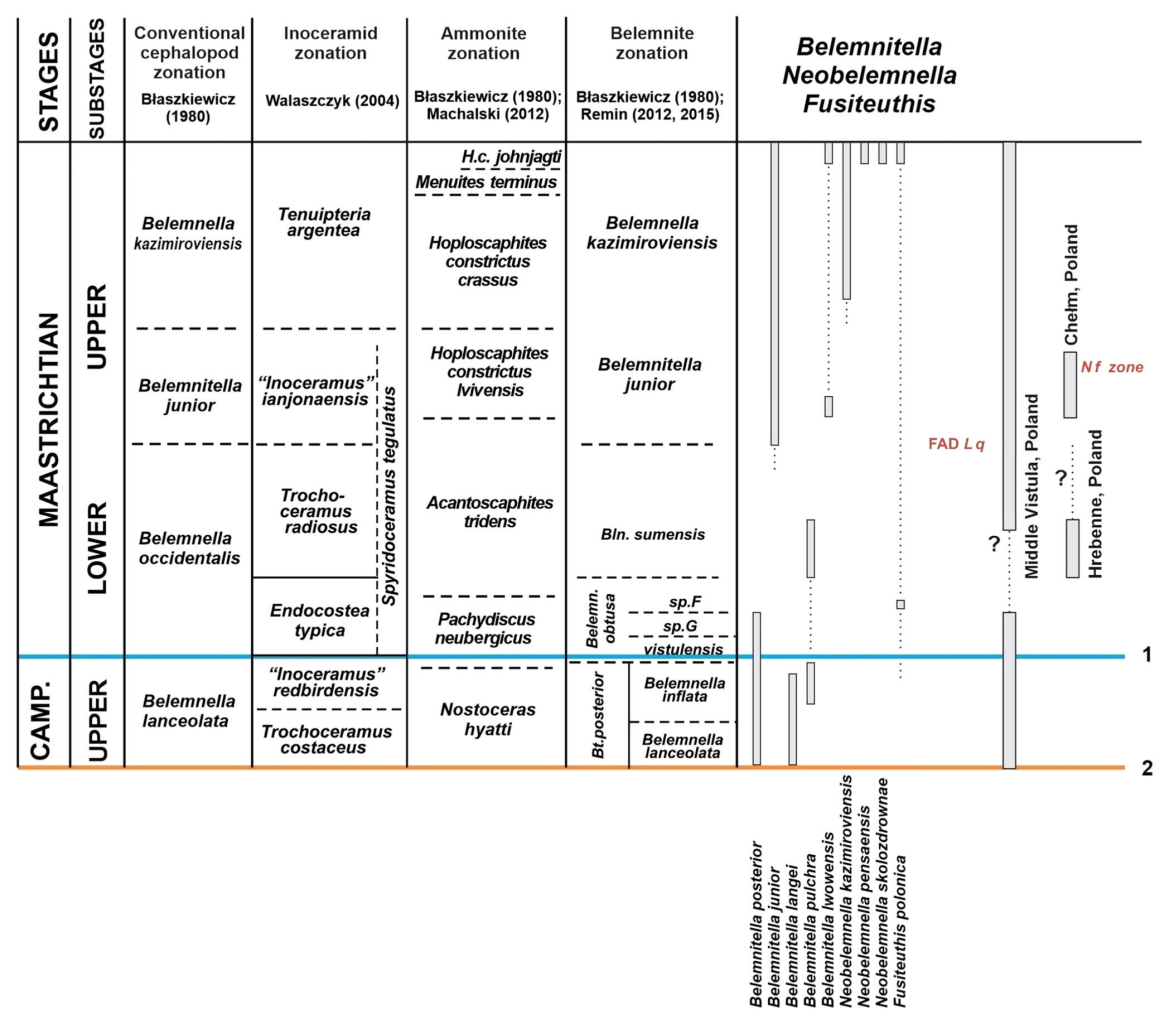
Stratigraphic ranges of species of *Belemnitella, Neobelemnella*, and *Fusiteuthis* and proposed belemnite zonation for the Middle Vistula valley section, central Poland; base of the Maastrichtian Stage according to the GSSP at Tercis, France (1) and as conventionally based on belemnites for the Boreal Realm (2). (L q: *Lithraphidites quadratus*; N f: *Nephrolithus frequens*).

Christensen (2000) distinguished two groups, the *Belemnitella mucronata-* and the *Belemnitella langei*-group. Both groups are present in the topmost Campanian *Belemnella lanceolata* and *Belemnella inflata* zone of the Middle Vistula valley (Fig. 3). Of the *mucronata*-group only *Belemnitella posterior* Kongiel, 1962 is present (Keutgen & Remin, unpublished). It resembles *Belemnitella carlsbergensis* Christensen, 1998 from the *lanceolata* Zone of Sweden, which may represent a junior synonym. Last representatives of *Belemnitella langei* Jeletzky, 1948 and records of *Belemnitella pulchra* Schulz, 1982, both members of the *langei*-group, are known from the Middle Vistula River valley as well (Keutgen & Remin, unpublished; Fig. 3). With up to three species of *Belemnitella*, the latest Campanian *Belemnella* zones of the Middle Vistula section are characterised by a comparatively high species diversity.

From the lower Maastrichtian *Belemnella obtusa* Zone only *Bt. posterior* is known from the Middle Vistula River valley (Remin, 2015), whereas from the United Kingdom, Belgium and the Netherlands the last representatives of *Belemnitella minor* II are recorded (Christensen, 1995, 1999; Keutgen & van der Tuuk, 1991), in addition to rare *Bt. pulchra* from Belgium (Christensen, 1999) and northern Germany (Schulz, 1982).

*Belemnitella* species from the lower Maastrichtian *Belemnella sumensis* Zone are rarely recorded and belong to *Belemnitella junior* Nowak, 1913 (Kongiel, 1962; Keutgen & van der Tuuk, 1991; Keutgen et al., 2010) and *Bt. pulchra* (Schulz, 1982; Remin, 2018).

The Upper Maastrichtian *Belemnitella junior* Zone is defined in practice either by the first appearance of *Bt. junior* or, in case the species first co-occurs with representatives of *Belemnella*, by the disappearance of representatives of the latter. From the *junior* Zone representatives of both the *mucronata*-group (*Bt. junior*) and the *langei*-group (*Belemnitella lwowensis* Naidin, 1952) are known (Fig. 3). *Bt. lwowensis* is regarded as a descendent of *Bt. pulchra*, but the stratigraphic level at which the transition between the two species took place is unclear. *Bt. lwowensis* and *Bt. junior* are sporadically recorded also from the latest Maastrichtian *Neobelemnella kazimiroviensis* Zone (Kongiel, 1962; Christensen et al., 2004). They became extinct together with all other belemnite species at the K-Pg boundary.

**Figure 4:**
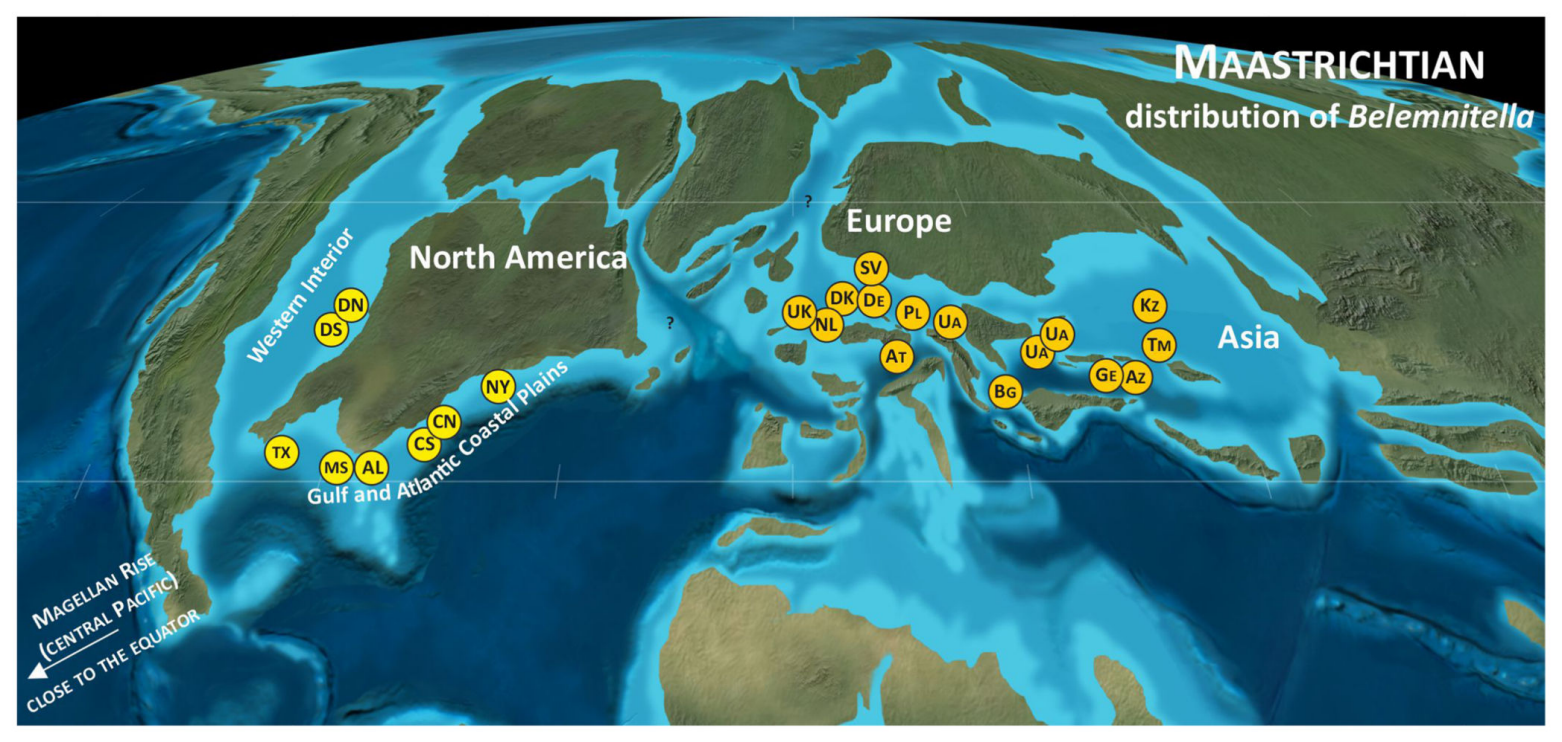
Paleogeographic distribution of the representatives of *Belemnitella*. Letter symbols are standard symbols for countries and states (US). Used with permission of Colorado Plateau Geosystems Inc.Global Paleogeography and Tectonics in Deep Time © 2016.

**Figure 5:**
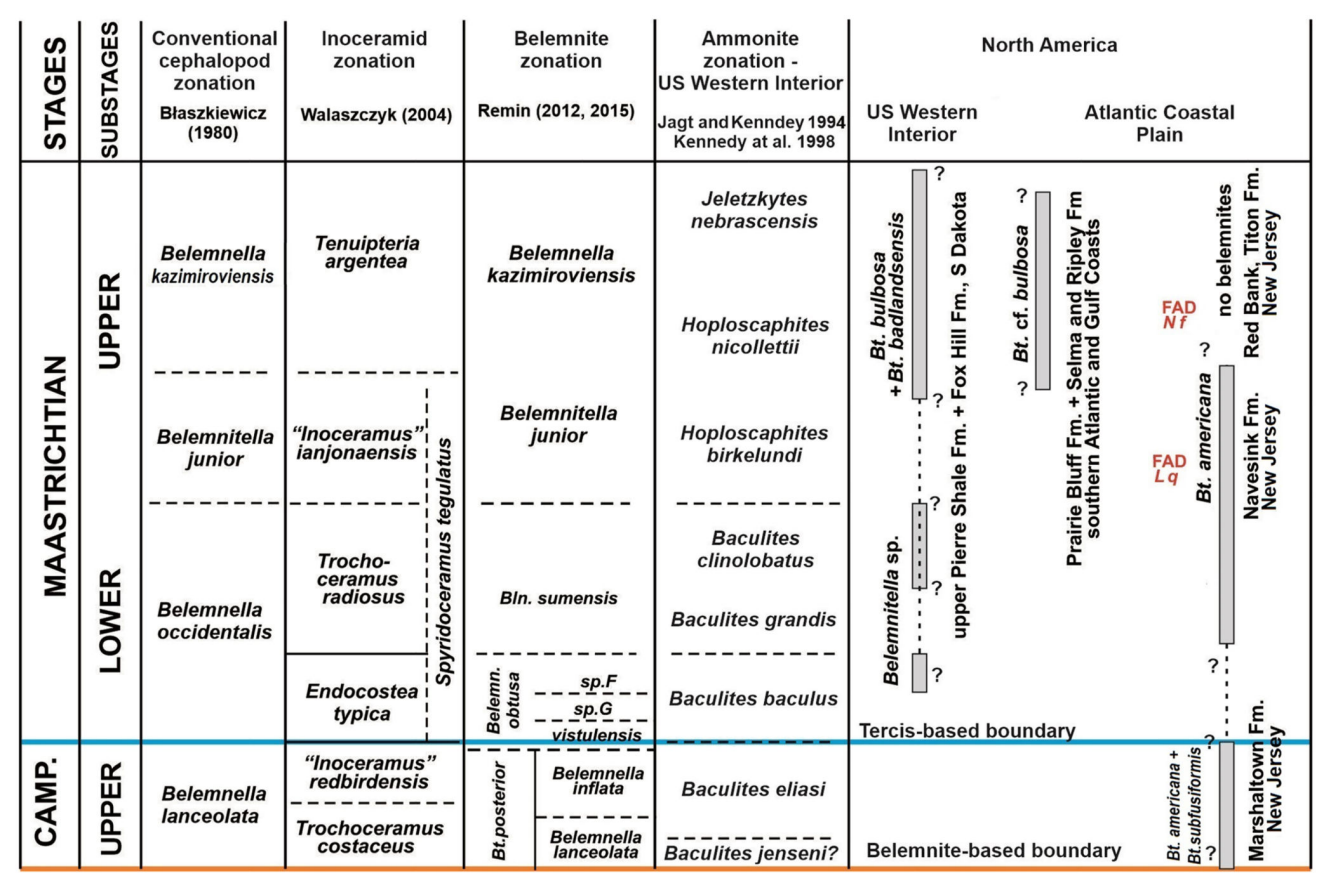
Ammonite, belemnite, and inoceramid bivalve stratigraphy for the topmost Campanian and Maastrichtian in selected sections in the US Western Interior and Gulf and Atlantic Coastal Plains. (L q: *Lithraphidites quadratus*; N f: *Nephrolithus frequens*).

*Belemnitella* was characterised by a wider geographical distribution than *Belemnella* (Figs. 2, 4). The former is also known from Maastrichtian deposits in North America, namely from the Atlantic Coastal Plain and the Western Interior. From the Monmouth County region of New Jersey, Remin in Kopun et al. (2012) identified *Belemnitella americana* (Morton, 1830) and *Belemnitella subfusiformis* (Whitfield, 1892) from the Navesink Formation, the latter species found only locally at the base of the Formation (Fig. 5). According to Sugarman et al. (1995), the base of the Navesink Formation is situated close to the base of the calcareous nannofossil zone CC25a (UC19). The FAD of *L. quadratus* (base UC20a) is assumed to be within the Navesink Formation, and the FAD of *N. frequens* (base UC20b) is already in the lower part of the Red Bank Formation and above the stratigraphic youngest records of *Bt. americana* in the Navesink Formation of New Jersey, indicating its disappearance in the lowermost upper Maastrichtian at least for the Monmouth County region, where the stratigraphic range of *Bt. americana* is best documented. For the Western Interior, Larson (2010) reported rare specimens of *Belemnitella* cf. *bulbosa* Meek & Hayden, 1857 (*Belemnitella* sp. of Kennedy et al., 1998) from the *Baculites baculus* and *Baculites clinolobatus* zones of the Pierre Shale of South Dakota (Fig. 5). The typical *Belemnitella bulbosa* Meek & Hayden (1857) is a rare species and regionally confined to the Fox Hills Formation and upper Pierre Shale of the Western Interior (South and North Dakota), occurring throughout the Timber Lake facies (*Jeletzkytes nebrascensis* Zone) and the Trail City facies (*Hoploscaphites nicolletii* Zone). Remin in Landman et al. (2013) distinguished two belemnite species in the Enning facies at Badlands National Park, South Dakota: *Bt. bulbosa* and *Belemnitella badlandsensis* Landman et al., 2013. Both species from the *nebrascensis* Zone differ mainly in the observed range of their fissure angles.

For the sake of completeness, it may be added that Zakharov et al. (2007, 2012) recorded *Belemnitella*? sp. from Late Campanian-Maastrichtian, likely mid-Maastrichtian deposits of the Magellan Rise in the Pacific Ocean.

### *Genus* Neobelemnella Naidin, 1975

**Figure 6:**
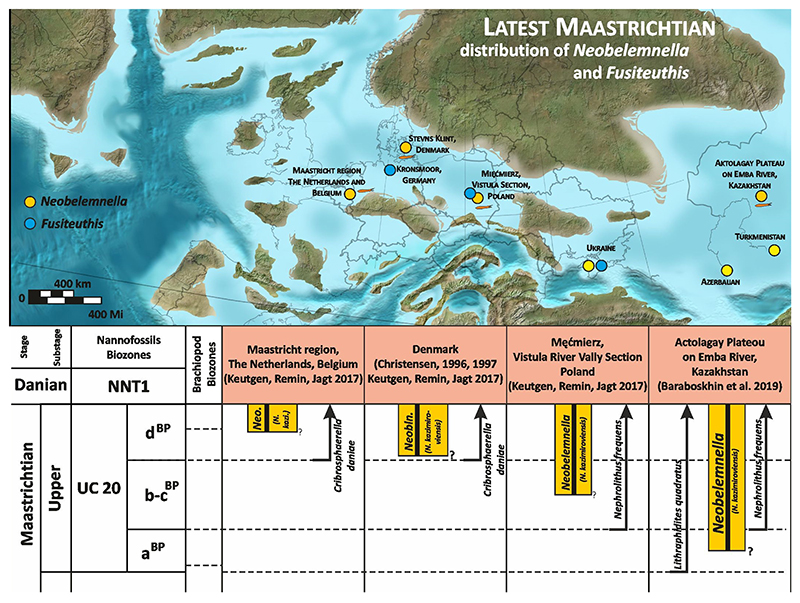
Paleogeographic distribution of the representatives of *Neobelemnella* and *Fusiteuthis* and the diachronic appearance of *Neobelemnella* across Europe against the FAD of selected nannoplankton species. Used with permission of Colorado Plateau Geosystems Inc.Global Paleogeography and Tectonics in Deep Time © 2016.

The genus *Neobelemnella* has recently been revised by Keutgen et al. (2017) and Keutgen & Keutgen (2020) applying different methodological approaches. *Neobelemnella kazimiroviensis* (Skołozdrówna, 1932) and *Neobelemnella skolozdrownae* (Kongiel, 1962) were recognised in both approaches. While the first species is widely recorded from Central Asia, Russia, Poland, Denmark, the Netherlands and Belgium, the second is limited to the uppermost Maastrichtian of Poland, Denmark, the Netherlands and Belgium (Fig. 6). *Neobelemnella* aff. *kazimiroviensis* (Skołozdrówna, 1932) was recorded only from Russia and Kazakhstan, while *Neobelemnella pensaensis* (Naidin, 1952) is difficult to separate from *N. kazimiroviensis* as transitory forms occur (Keutgen & Keutgen, 2020).

### *Genus* Fusiteuthis Kongiel, 1962

Single representatives of this dubious genus with the single species *Fusiteuthis polonica* Kongiel, 1962 have been reported from the uppermost Campanian to upper upper Maastrichtian in northwest Europe and the Crimea: from the *lanceolata* Zone of Kronsmoor (northern Germany), the *obtusa* Zone of Dziurków (Poland) and from the uppermost Maastrichtian of Crimea and Poland (Christensen, 2002; Remin, 2010). Remin (2010) suggested that *F. polonica* could represent an intergeneric hybrid between the genera *Belemnitella* and *Belemnella* or *Belemnitella* and *Neobelemnella* since it possesses features characteristic for both study genera.

**Figure 7:**
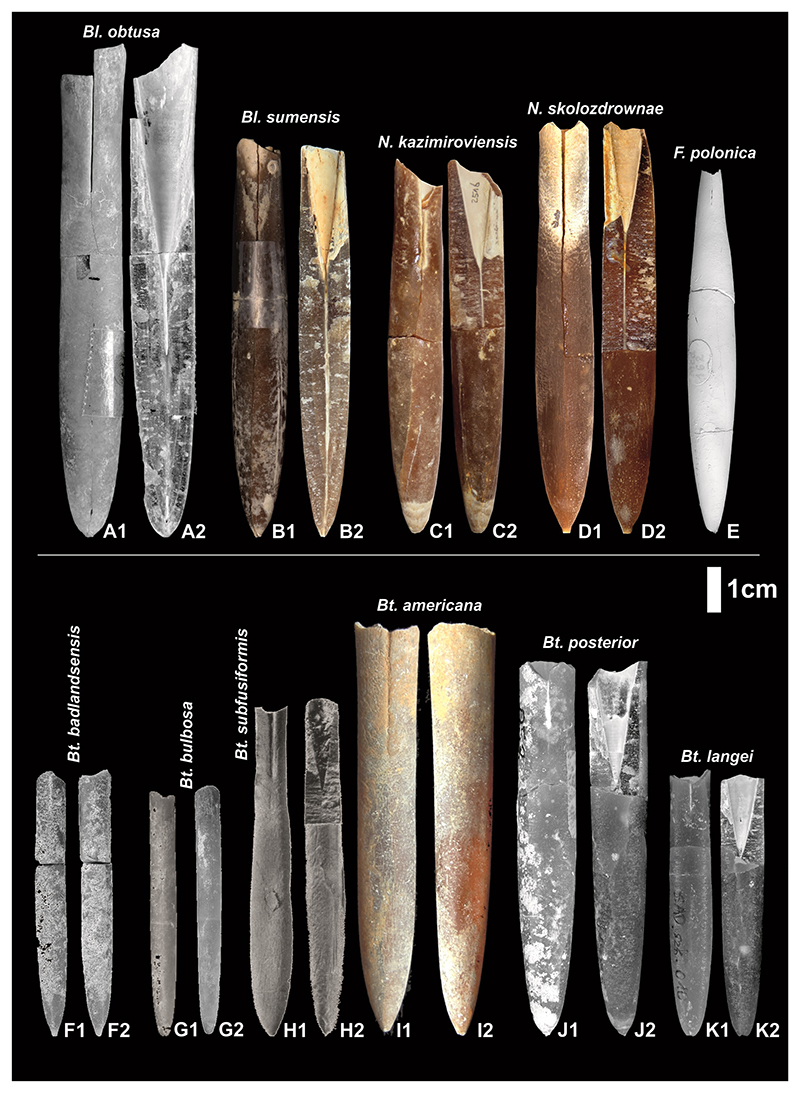
A1-A2. *Belemnella obtusa* Schulz, 1979, KN 810 − holotype, Kronsmoor, Germany (Schulz, 1979; Remin, 2012); B1-B2. *Belemnella sumensis* Jeletzky, 1949, HR014, Hrebenne, Poland (Remin, 2018); C1-C2. *Neobelemnella kazimiroviensis* (Skołozdrówna, 1932), NHMM MK 2516, Maastricht, The Netherlands (Keutgen et al., 2017); D1-D2. *Neobelemnella skolozdrownae* (Kongiel, 1962), MWGUW ZI/69/70, Nasiłów, Poland (Keutgen et al., 2017); E1. *Fusiteuthis polonica* Kongiel, 1962, Mcd 162 - holotype, Nasiłów, Poland (Kongiel, 1962); F1-F2. *Belemnitella badlandsensis* Landman, et al., 2013, AMNH 79950, holotype, Fox Hills Formation, AMNH loc. 3283, Badlands National Park, Pennington County, South Dakota (Landman et al., 2013); G1-G2. *Belemnitella bulbosa* Meek & Hayden, 1857, AMNH 79945, Fox Hills Formation, AMNH loc. 3283, Badlands National Park, Pennington County, South Dakota (Landman et al., 2013); H1-H2. *Belemnitella*/?*Neobelemnella subfusiformis* (Whitfield, 1892), ANSP 19488, holotype, New Jersey (Jeletzky, 1962); I1-I2. *Belemnitella americana* (Morton, 1830), (ex Remin’s coll.), New Jersey; J1-J2. *Belemniella posterior* Kongiel, 1962, Sad/szk/053, Vistula section, Poland (Remin, 2015); K1-K2.

Typical belemnite species of the here mentioned genera are shown in Fig. 7, indicating the variation in size and shape of the genera.

## Discussion

In the uppermost Campanian, the genus *Belemnella* appeared almost contemporaneously from Kazakhstan in the east to the United Kingdom in the west (Fig. 1) and rapidly began to evolve into local (sub)species, thereby replacing the genus *Belemnitella*. With respect to the genus *Belemnitella*, species diversity was unusually high with two species in the uppermost Campanian *lanceolata* Zone of the Middle Vistula River valley (Poland), which is in contrast to only one species of *Belemnella* (Remin, 2012, 2015). In the latest Campanian *inflata* Zone, the number of *Belemnitella* species rose to three and that of *Belemnella* to four, resulting in a very unusual belemnite species diversity of seven. However, the number of collected *Belemnitella* specimens (*N* = 22) is distinctly smaller (35%) than that of *Belemnella* (*N* = 40; Remin, 2012, 2015). The spread of *Belemnella* is associated with the negative excursion in δ^13^C at the Campanian-Maastrichtian boundary (Wilmsen et al., 2019), which is usually interpreted as indicative of a sea-level fall. An argument in favour of this interpretation represents the earliest Maastrichtian isotope minimum (eMim) dated by Wilmsen et al. (2019) at 71.94 Myr, which corresponds well with the sea-level lowstand KMa1 of Haq (2014) at 72.0 Myr. Following Wiese’s et al. (2009) argumentation, the sea-level fall progressively created conditions for the migration of *Belemnella* by the expansion of shelf settings with favourable depths for belemnite immigration. However, the origin of the genus *Belemnella* is still a mystery. Schulz (1979) suggested a relationship with the genus *Belemnellocamax* Naidin, 1964b, the latest known representative of which being almost exclusively recorded from the Baltoscandian Subprovince. By contrast, Naidin (1974) supported an origin from eastern European species of *Belemnitella*. The appearance of *Belemnella* also coincided with a conspicuous latest Campanian cooling event, which has received considerable attention (e.g. Linnert et al., 2014, 2016; Thibault et al., 2015; Wilmsen & Niebuhr, 2017) and suggests an immigration of *Belemnella* from a cooler northern or north-eastern region.

The increase in the number of *Belemnella* species within *c*. 800 kyr in the *lanceolata* and *inflata* zones may be interpreted as a typical example for adaptive radiation, a process where organisms diversify rapidly from an ancestral species into a multitude of new forms due to a change in the environment or the availability of new ecological niches making new resources available. This suggests first of all parapatric and peripatric speciation, both of which go hand in hand with adaptation to ecological niches. Allopatric speciation is also taken into consideration, for example, when populations become separated by a geographic barrier or simply by a large distance between them. If, however, a reproductive barrier is removed, reuniting two previously isolated populations, fertile hybrids may occur. As a consequence, the two populations may unite again, yet exhibiting a larger range of variation. Alternatively, hybrids may be of low fitness, reinforcing the separation of the two populations and resulting in speciation. In rare cases, new species can also be created through hybridization, followed by reproductive isolation, if the hybrids are favoured by natural selection. All of these different speciation processes could have led to the evolution of different ‘morphotypes’ such as distinguished in the Kronsmoor quarry ‘Saturn’ and the Middle Vistula River valley (Remin in Niebuhr et al., 2011; Remin, 2012).

In the Middle Vistula valley, the Campanian-Maastrichtian boundary was crossed by only two of seven species, one each belonging to *Belemnitella* and *Belemnella*, indicating the severe impact of a hypothetical ‘boundary event’ on the belemnite populations (Figs. 1, 3). From The Middle Vistula area, only a single species of the *mucronata*-group (*Bt. posterior*) is recorded. From Western Europe (United Kingdom, Netherlands, Belgium), a second species of this group is recorded (*B. minor* II), and a representative of the *langei*-group (*Bt. pulchra*) is known from the *obtusa* Zone of Kronsmoor in northern Germany and the Mons Basin, Belgium (Schulz, 1982; Christensen, 1999).

In the Middle Vistula section, this ‘boundary event’ corresponds to a characteristic lithologic unit, the ‘boundary marl’. Within the ‘boundary marl’ as well as directly below and above this level, belemnites are rare or absent (Remin, 2012), altogether indicating that the ‘boundary marl’ seems thus to represent an interval in which an important event in the early evolutionary history of *Belemnella* and *Belemnitella* took place. At the same stratigraphic level in the ‘Saturn’ quarry (Kronsmoor) representatives of *Belemnella* are lacking, immediately below the Campanian-Maastrichtian boundary at mB609 (Niebuhr et al., 2011). Both *Bl. obtusa* and *Bl. vistulensis* have their FAD somewhat above the Campanian-Maastrichtian boundary, while *Bl*. sp. A and *Bl*. sp. F successively appeared in Poland and northern Germany during the following *c*. 600 kyr, while *Belemnitella* became rare, still resulting in the co-existence of up to six belemnite species in the *obtusa* Zone compared to seven in the topmost Campanian of the Middle Vistula valley (Figs. 1, 3). The percentage of *Belemnitella* in the fauna comprises *c*. 7%.

The available data indicate that the ‘boundary marl’ event did not favour *Belemnella* over *Belemnitella*, but hit both, which manifests itself in the species composition and frequency. It remains to be tested to what extent the ‘boundary marl’ event was a supra-regional event, but it obviously affected belemnite evolution in northern Germany and Poland.

From the *sumensis* Zone of Poland in total three species of *Belemnella* and two of *Belemnitella* are recorded (Figs. 1, 3), whereby the first record of the second species *Bt. junior* is from a level high in the *sumensis* Zone at Boiska (Kongiel, 1962). From the *sumensis* Zone at Hrebenne (southeast Poland), 27 specimens were studied, only a single (4%) belonging to *Bt. pulchra*.

In the type Maastricht region (the Netherlands, Belgium), the stratigraphically oldest *Bt. junior* is known from Altembroeck (northeast Belgium) from a level close to the base of unit 4 of the Vijlen Member (*sumensis* Zone), which was estimated *c*. 300 kyr older than the base of the Vijlen Member as exposed at Hallembaye quarry (Belgium, 50°44’54” N, 5°38’54” E), formerly known as Ciment Portland Liégeois (CPL), currently Kreco (Keutgen, 2018). Vellekoop et al. (2022, table 1) dated the base of the Vijlen Member at Hallembaye at *c*. 70.4 Myr, which implies for the base of the unit 4 of the Vijlen Member at Altembroeck a numerical age of *c*. 70.7 Myr. An alternative interpretation would take the dating of the ‘Zonneberg Horizon’ by Vellekoop et al. (2022, table 1) as a reference, which would suggest a numerical age for the base of unit 4 of the Vijlen member of 70.3 Myr (= 69.9 + 0.4 Myr), assuming a sedimentation period for the units 4 and 5 of the Vijlen Member of 400 kyr (Keutgen, 2018). Although these calculations allow only a rough estimate for the FAD of *Bt. junior* at Altembroeck between *c*. 70.3 and 70.7 Myr, correlation with the *sumensis/tridens* isotope minimum, dated at 71.1 Myr (Wilmsen et al., 2019), might be plausible when taking into account that deposition of unit 4 of the Vijlen Member occurred during a minor sea-level lowstand (Felder & Bless, 1994). Thibault et al. (2012, fig. 8) correlated the level of the *sumensis/tridens* isotope minimum with a position within the carbon-isotope events M1- (*c*. 70.2−71.0 Myr), which would imply a slightly younger age than that suggested by Wilmsen et al. (2019). A rough correlation of the base of unit 4 of the Vijlen Member at Altembroeck with the *sumensis/tridens* isotope minimum at Kronsmoor is also supported by records of *Bl. praearkhangelskii* above this level at both localities (Schulz, 1979; Keutgen, 1997). If the correlation of the appearance of *Bl. praearkhangelskii* in Altembroeck and Kronsmoor and that of the *sumensis/tridens* isotope minimum and the base of unit 4 of the Vijlen Member can be confirmed in future studies, this would imply that the appearance of both *Bt. junior* and *Bl. praearkhangelskii* would have been supported by a sea-level fall that created conditions favourable for the immigration of belemnites (Wiese et al., 2009). Noteworthy, the appearance of *Bt. junior* in the Maastricht area did not coincide with a temperature increase (Vonhof et al., 2011; Keutgen, 2018).

The stratigraphically oldest record of *Bt. junior* in the Maastricht area is conservatively dated at *c*. 70.3 Myr. It suggests a concurrent range of *Bl. sumensis* and *Bt. junior* in the Maastricht region of *c*. 250 kyr (Keutgen, 2018). The disappearance of the genus *Belemnella* in the Maastricht region thus corresponds to a position in the ‘upper’ *sumensis* Zone in northern Germany, indicating that the genus *Belemnella* disappeared as much as *c*. 1.6 Myr earlier in the type Maastricht area than in northern Germany (Thibault et al., 2012).

At Boiska, Poland, *Bt. junior* and *Bl. sumensis* co-occur as well, however, at a stratigraphically higher level as indicated by the presence of *L. quadratus* (Dubicka & Peryt, 2012). Seemingly, the situation at the Aktolagai plateau section (middle course of the Emba River, Western Kazakhstan) around the FAD of *L. quadratus* resembles that at Boiska. Baraboshkin et al. (2019, fig. 6) reported ?*Bt. junior* co-occurring with *Belemnella*, probably with *Bl. sumensis*. Already Naidin (1973) reported on the co-occurrence of *Bt. junior* and *Bl. sumensis* from the northern part of the Donbass region. By contrast, *Bt. junior* is not known from the northeastern parts of the Russian Platform, for example, from the Volga region (Naidin, 1973).

In the lower upper Maastrichtian at least in Western and Central Europe, the number of belemnite species is further reduced to two, both belonging to *Belemnitella* (Christensen et al., 2004; Remin in Dubicka & Peryt, 2011). The cause of the extinction of *Belemnella* in these areas is unclear, but might be linked to the onset of the early Maastrichtian warming. Vonhof et al. (2011, table 1) recorded oxygen isotope data (δ^18^O) from the type Maastrichtian area (Netherlands) measured almost exclusively at belemnite guards with the metre scale of the collected belemnites set at 0 at the Zonneberg Horizon, which was interpreted by the late P.J. Felder as the base of unit 6 of the Vijlen Member (Keutgen, 2018). It follows from their data that δ^18^O-values remained relatively stable until close to the top of unit 5 of the Vijlen Member and then decreased to a lower level that was reached close to the boundary between the Lixhe 1 and Lixhe 2 Members. Thus, temperature started to rise in the Maastricht region approximately at *c*. 70.0−70.4 Myr, and *Belemnella* disappeared *c*. 50−100 kyr earlier, somewhat above the base of unit 5 of the Vijlen Member − stratigraphically younger records of *Belemnella* are considered reworked (Keutgen, 2018). This would favour a close relationship between temperature rise and demise of *Belemnella*. By contrast, the disappearance of *Belemnella* in northern Germany (Hemmoor quarry) is dated *c*. 68.4 Myr (Schulz, 1979; Thibault et al., 2012), but for the Stevns-1 borehole (Denmark), which may be best comparable to Hemmoor, the temperature increase appeared at *c*. 69.5 Myr (Thibault et al., 2016), *c*. 1.1 Myr earlier than the demise of *Belemnella*. Different to the belemnite-derived δ^18^O-values from the Maastricht region, those from the Stevns-1 borehole represent bulk samples. While the measurements of δ^18^O-values from belemnites reflect the temperature, where the belemnites lived (Zakharov et al., 2014), bulk samples relate to the sea-surface temperature (Thibault et al., 2016) and may not reflect the temperature conditions of the preferred biotope of *Belemnella*. Wilmsen & Niebuhr (2017) suggested a nektobentic mode of life for belemnites and Keutgen et al. (2017) assumed that small (juvenile) belemnites might have preferred a shallower-marine habitat, while adults ventured out into open and deeper water. Hoffmann and Stevens (2020) pointed out that long lateral or vertical migration of belemnites of the Campanian genera *Gonioteuthis* Bayle, 1878 and *Belemnitella* d’Orbigny, 1840 are less probable, as indicated by the presence of nearly all ontogenetic growth stages in populations collected from the marly limestones and calcareous marls exposed in the Höver quarry (northern Germany). These sediments were deposited at depths less than that of the typical Chalk facies but distinctly below storm-wave base (Christensen, 2000), with a depth of *c*. 70−110 m calculated from the data of Wilmsen & Niebuhr (2017). It is suggested that older belemnites could have escaped the warmer sea-surface temperatures recorded by Thibault et al. (2016) at the bottom of the mid Maastrichtian northern German shelf sea with a water depth of 100−150 m (Wilmsen & Niebuhr, 2017), being deeper than the Vijlen Chalk Member, for example, at Hallembaye with a water depth of *c*. 80 m (Jagt & Jagt-Yazykova, 2012). Assuming a temperature gradient of 12.5−18.75 m/1°C, Wilmsen & Niebuhr (2017) would explain a by *c*. 2.0−3.5°C lower temperature at the bottom of the Hemmoor Chalk Sea. However, it may be hypothesised that juvenile representatives of *Belemnella* may have been exposed to higher temperatures as a consequence of their preferred shallow marine habitat. It is assumed that either they successfully adapted to warmer temperature or that their spawning grounds were situated in colder regions, presumably towards the north.

With the appearance of the genus *Neobelemnella* in the upper upper Maastrichtian, species diversity increased and the number of species rose to up to five in the Middle Vistula valley − three belonging to *Neobelemnella* (Keutgen & Keutgen, 2020) and two to *Belemnitella* (Kongiel, 1962; Christensen et al., 2004), the latter known only from sporadic records. Rare finds of *Fusiteuthis polonica* are not considered.

The stratigraphically oldest records of *N. kazimiroviensis* are from Kazakhstan (Aktolagay Plateau) from the calcareous nannofossil zone UC20a, above the FAD of calcareous nannofossil *L. quadratus*, but below the FAD of *N. frequens* (Baraboshkin et al., 2019). In Poland, the stratigraphically oldest representatives are from Mięćmierz, which is situated above the FAD of *N. frequens* (Dubicka & Peryt, 2012) and, hence, they are considered stratigraphically younger than those from Kazakhstan (Fig. 6). First records of *N. kazimiroviensis* from Denmark are even younger (*Cribrosphaerella daniae* calcareous nannofossil zone) than those from Poland (Machalski, 1996), and specimens from the Maastricht region (the Netherlands, Belgium) appear even later (*c*. 50−100 kyr before the K-Pg boundary), altogether implying a westwards migration of this species during the upper Maastrichtian.

## References

[R1] Baraboshkin EY, Benyamovskiy VN, Guzhikov AY, Aleksandrova GN, Pervushov EM, Celler WB, Ovechkina MN, Kalyakin EA, Kopaevich LF, Guzhikova AA, Pokrovskiy BG, Zholtaev GZ, Ali-Zade AA, Degtyarev KE, Ergaliev GK, Kilipko VA, Sakiev KS, Seitmuratova EY, Zhaymina VY, Fazylov EM, Bekenova GK, Musina ES (2019). Integrated study of Campanian/Maastrichtian boundary interval at Volga region (Russia) and Aktolagay Plateau (West Kazakhstan) of the Russian Platform.

[R2] Baraboshkin EY, Guzhikov AY, Aleksandrova GN, Akinin VV, Ryabov IP, Ustinova MA, Rtischev NA, Vishnevskaya VS (2024). Reference section of the Campanian stage of the Southwestern Crimea: problems of substage subdivision and global correlation. Stratigraphy and Geological Correlation.

[R3] Baraboshkin EY, Guzhikov AY, Aleksandrova GN, Fomin VA, Pokrovskiy BG, Grishchenko VA, Manikin AG, Naumov EV (2021). New sedimentological, magnetostratigraphic, and biostratigraphic data on the Campanian-Maastrichtian of Beshkosh Mountain, Southwest Crimea. Stratigraphy and Geological Correlation.

[R4] Bayle E (1878). Fossiles principaux des terrains de la France. Explication de la Carte Geologique de la France.

[R5] Boussaha M, Thibault N, Stemmerik L (2016). Integrated stratigraphy of the late Campanian-Maastrichtian in the Danish Basin: Revision of the Boreal calcareous nannofossil zonation. Newsletters on Stratigraphy.

[R6] Bramlette MN, Martini E (1964). The great change in calcareous nannoplankton fossils between Maestrichtian and Danian. Micropaleontology.

[R7] Christensen WK (1975). Upper Cretaceous belemnites from the Kristianstad area in Scania. Fossils and Strata.

[R8] Christensen WK (1995). *Belemnitella* from the Upper Campanian and Lower Maastrichtian chalk of Norfolk. England Special Papers in Palaeontology.

[R9] Christensen WK (1997a). Palaeobiogeography and migration in the Late Cretaceous belemnite family Belemnitellidae. Acta Palaeontologica Polonica.

[R10] Christensen WK (1997b). The Late Cretaceous belemnite family Belemnitellidae: Taxonomy and evolutionary history. Bulletin of the Geological Society of Denmark.

[R11] Christensen WK (1998). *Belemnitella* from the lowermost Maastrichtian of Scania, southern Sweden. Bulletin of the Geological Society of Denmark.

[R12] Christensen WK (1999). Upper Campanian and Lower Maastrichtian belemnites from the Mons Basin. Belgium. Bulletin de l’Institut royal des Sciences Naturelles de Belgique.

[R13] Christensen WK (2000). *Belemnitella schulzi* sp. nov. from the uppermost Campanian and lowest Maastrichtian chalks of northwest Germany and Denmark. Acta Geologica Polonica.

[R14] Christensen WK (2002). *Fusiteuthis polonica*, a rare and unusual belemnite from the Maastrichtian. Acta Palaeontologica Polonica.

[R15] Christensen WK, Schmid F, Schulz M-G (2004). *Belemnitella* from the Upper Maastrichtian of Hemmoor, Northwest Germany. Geologisches Jahrbuch.

[R16] Dubicka Z, Peryt D (2011). Integrated biostratigraphy of Upper Maastrichtian chalk at Chełm (SE Poland). Annales Societatis Geologorum Poloniae.

[R17] Dubicka Z, Peryt D (2012). Latest Campanian and Maastrichtian palaeoenvironmental changes: implications from an epicontinental sea (SE Poland and western Ukraine). Cretaceous Research.

[R18] Dubicka Z, Wierny W, Bojanowski MJ, Rakociński M, Walaszczyk I, Thibault N (2023). Multi-proxy record of the mid-Maastrichtian event in the European Chalk Sea: paleoceanographic implications. Gondwana Research.

[R19] Felder PJ, Bless MJM (1994). The Vijlen Chalk (early Early to early Late Maastrichtian) in its type area around Vijlen and Mamelis (southern Limburg, The Netherlands). Annales de la Société géologique de Belgique.

[R20] Górka H (1957). Les Coccolithophoridés du Maestrichtien supérieur de Pologne. Acta Palaeontologica Polonica.

[R21] Haq BU (2014). Cretaceous eustasy revisited. Global and Planetary Change.

[R22] Hoffmann R, Stevens K (2020). The palaeobiology of belemnites − foundation for the interpretation of rostrum geochemistry. Biological Reviews.

[R23] Jagt JWM, Jagt-Yazykova EA, Jagt JWM, Donovan SK, Jagt-Yazykova EA (2012). Stratigraphy of the type Maastrichtian - a synthesis, Fossils of the type Maastrichtian (Part 1). Scripta Geologica Special Issue.

[R24] Jeletzky JA (1941). Doklady Akademii Nauk SSSR.

[R25] Jeletzky JA (1948). Zur Kenntnis der Oberkreide der Dnjepr-Donetz-Senke und zum Vergleich der russischen borealen Oberkreide mit derjenigen Polens und Nordwesteuropas. Geologiska Föreningen i Stockholm Förhandlingar.

[R26] Jeletzky JA (1949). Über den taxonomischen Wert einiger morphologische Elemente des Rostrums der belemnitellenartigen Formen (Familie Belemnitellidae Pavlow, 1913), sowie über die Gattung *Belemnella* (Nowak, 1913, subg.) Jeletzky, 1941, ihre Phylogenie und einige Vertreter. Neues Jahrbuch für Mineralogie, Geologie und Paläontologie, Monatshefte.

[R27] Jeletzky JA, Richards HG, Ramsdell RC (1962). Cretaceous belemnites of New Jersey. The Cretaceous Fossils of New Jersey. Paleontology Series, State of New Jersey, Department of Conservation and Economic: Development.

[R28] Kennedy WJ, Landman NH, Christensen WK, Cobban WA, Hancock JM (1998). Marine connections in North America during the late Maastrichtian: palaeogeographic and palaeobiogeographic significance of *Jeletzkytes nebrascensis* Zone cephalopod fauna from the Elk Butte Member of the Pierre Shale. SE South Dakota and NE Nebraska Cretaceous Research.

[R29] Keutgen N (1997). *Belemnella* (Belemnella) cf. *praearkhangelskii* Naidin, 1964 from the Vijlen Member at Altembroeck (NE Belgium, Early Maastrichtian). Geologie en Mijnbouw.

[R30] Keutgen N (2018). A bioclast-based astronomical timescale for the Maastrichtian in the type area (southeast Netherlands, northeast Belgium) and stratigraphic implications: the legacy of P.J. Felder. Netherlands Journal of Geosciences.

[R31] Keutgen N, Jagt JWM, Felder PJ, Yazykova E (2010). Stratigraphy of the upper Vijlen Member (Gulpen Formation; Maastrichtian) in northeast Belgium, the southeast Netherlands and the Aachen area (Germany), with special reference to belemnitellid cephalopods. Netherlands Journal of Geosciences.

[R32] Keutgen N, Keutgen AJ (2020). Belemnites of the *Neobelemnella kazimiroviensis* group − a comparison of the self-organizing Kohonen networks algorithm with the classic palaeopopulation statistical approach and approaching their origin. Neues Jahrbuch für Geologie und Paläontologie - Abhandlungen.

[R33] Keutgen N, Remin Z, Jagt JWM (2017). The late Maastrichtian *Belemnella kazimiroviensis* group (Cephalopoda, Coleoidea) in the Middle Vistula valley (Poland) and the Maastricht area (the Netherlands, Belgium) − taxonomy and palaeobiological implications. Palaeontologia Electronica.

[R34] Keutgen N, Remin Z, Walaszczyk IP (2012). Early representatives of the belemnite genus *Belemnella* (Cephalopoda) from the uppermost Campanian-Lower Maastrichtian of the Middle Vistula River section, central Poland. Acta Geologica Polonica.

[R35] Keutgen N, Van der Tuuk LA (1991). Belemnites from the Lower Maastrichtian of Limburg, Aachen and Liège. Mededelingen van de Rijks Geologische Dienst.

[R36] Kongiel R (1962). On belemnites from the Maastrichtian, Campanian and Santonian sediments in the Middle Vistula valley (Central Poland). Prace Muzeum Ziemi.

[R37] Kopun J, Castro C, Garb MP, Larina E, Remin Z (2012). Taphonomic analysis of a Late Cretaceous oyster bed at the top of the Mount Laurel Formation in Monmouth country New Jersey: Long term hiatal concentration, storm deposit or tsunami?.

[R38] Landman NH, Remin Z, Garb MP, Chamberlain JA (2013). Cephalopods from the Badlands National Park area, South Dakota: Reassessment of the position of the Cretaceous/Paleogene boundary. Cretaceous Research.

[R39] Larson NL (2010). Fossil Coleoids from the Late Cretaceous (Campanian & Maastrichtian) of the Western Interior.

[R40] Linnert C, Robinson SA, Lees JA, Bown PR, Pérez-Rodríguez I, Petrizzo MR, Falzoni F, Littler K, Arz JA, Russell EE (2014). Evidence for global cooling in the Late Cretaceous. Nature Communications.

[R41] Linnert C, Engelke J, Wilmsen M, Mutterlose J (2016). The impact of the Maastrichtian cooling on the marine nutrient regime − Evidence from midlatitudinal calcareous nannofossils. Paleoceanography.

[R42] Linnert C, Engelke J, Wilmsen M, Mutterlose J (2019). Environmental footprints of the early Maastrichtian cooling − The record of benthic foraminifera from northern Germany. Cretaceous Research.

[R43] Machalski M (1996). Scaphitid ammonite correlation of the Late Maastrichtian deposits in Poland and Denmark. Acta Palaeontologica Polonica.

[R44] Meek FB, Hayden FV (1857). Descriptions of new species of Gastropoda and Cephalopoda from the Cretaceous formations of Nebraska Territory. Proceedings of the Academy of Natural Sciences of Philadelphia.

[R45] Morton SG (1830). Synopsis of the organic remains of the Ferruginous Sand Formation of the United States, with geological remarks. American Journal of Science and Arts.

[R46] Naidin DP (1952). Verkhnemelovye belemnity zapadnoj Ukrainy. Trudy Moskovskogo Geologo-Razvedochnogo Instituta imemi S Ordzhinikidze.

[R47] Naidin DP (1964a). Byulleten’ Moskovskogo Obshchestva Ispytatelej Prirody, Otdel geologicheskii.

[R48] Naidin DP (1964b). Upper Cretaceous belemnites from the Russian Platform and adjacent areas. Actinocamax, Gonioteuthis, Belemnellocamax. Izdatelstvo Moskovskogo Gosudarstvennogo Universiteta, Moscow. Byulleten’ Moskovskogo Obshchestva Ispytatelei Prirody.

[R49] Naidin DP (1973). Byulleten Moskovskogo Obshchestva Ispytatelei Prirody Otdel Geologicheskii.

[R50] Naidin DP, Krumgol’ts GYa (1974). Subclass Endocochlia − Order Belemnitidae. Atlas of the Upper Cretaceous fauna of Donbas Moscov (Nedra).

[R51] Naidin DP, Menner WW, Moskvin MM, Naidin DP, Solovyev AN, Shimansky VN (1975). Late Maastrichtian belemnitellids of Eurasia. Evolution and change of the organic kingdom at the Mesozoic-Cainozoic boundary, Nauka (Moskva).

[R52] Niebuhr B, Hampton MJ, Gallagher LT, Remin Z (2011). Integrated stratigraphy of the Kronsmoor section (northern Germany), a reference point for the base of the Maastrichtian in the Boreal Realm. Acta Geologica Polonica.

[R53] Nowak J (1913). Untersuchungen über die Cephalopden der oberen Kreide in Polen. III Teil. Bulletin de l’Académie des Sciences de Cracovie. Classe des Sciences Mathématique et Naturelles. Série B Sciences Naturelles, for 1913.

[R54] Odin GS, Lamaurelle MA (2001). The global Campanian-Maastrichtian stage boundary. Episodes.

[R55] d’Orbigny A (1840). Terrains Crétacés (Paris).

[R56] Perch-Nielsen K (1973). Neue Coccolithen aus dem Maastrichtien von Dänemark, Madagaskar und Ägypten. Bulletin of the Geological Society of Denmark.

[R57] Peryt D, Dubicka Z, Wierny W (2022). Planktonic foraminiferal biostratigraphy of the Upper Cretaceous of the Central European Basin. Geosciences.

[R58] Remin Z (2010). *Fusiteuthis polonica* - an intergeneric belemnite hybrid − discussion.

[R59] Remin Z (2012). The *Belemnella* stratigraphy of the Campanian-Maastrichtian boundary: a new methodological and taxonomic approach. Acta Geologica Polonica.

[R60] Remin Z (2015). The *Belemnitella* stratigraphy of the Upper Campanian-basal Maastrichtian of the Middle Vistula section, central Poland. Geological Quarterly.

[R61] Remin Z (2018). Understanding coleoid migration patterns between eastern and western Europe-belemnite faunas from the upper lower Maastrichtian of Hrebenne, southeast Poland. Cretaceous Research.

[R62] Remin Z, Cyglicki M, Niechwedowicz M (2022a). Deep vs. shallow − two contrasting theories? A tectonically activated Late Cretaceous deltaic system in the axial part of the Mid-Polish Trough: a case study from southeast Poland. Solid Earth.

[R63] Remin Z, Krzywiec P, Stachowska A, Walaszczyk I, Todes J (2022b). Field trip Guides Faculty of Geology.

[R64] Schulz M-G (1979). Morphometrisch-variationsstatistische Untersuchungen zur Phylogenie der Belemniten-Gattung *Belemnella* im Untermaastricht NW Europas. Geologisches Jahrbuch.

[R65] Schulz M-G (1982). Erster Nachweis der Belemnitengattung *Belemnitella* (*B. pulchra* n. sp.) im mittleren Untermaastricht NW-Deutschlands. Geologisches Jahrbuch.

[R66] Skołozdrówna Z (1932). Znaczenie alveoli i szczeliny alveolarnej dla systematyki rodzaju *Belemnitella*. Posiedzenia Naukowe, Państwowego Instytutu Geologicznego.

[R67] Sugarman PJ, Kenneth GM, Bukry G, Feigenson MD (1995). Uppermost Campanian-Maestrichtian strontium isotopic, biostratigraphic, and sequence stratigraphic framework of the New Jersey Coastal Plain. GSA Bulletin.

[R68] Thibault N (2016). Calcareous nannofossil biostratigraphy and turnover dynamics in the late Campanian-Maastrichtian of the tropical South Atlantic. Revue de micropaléontologie.

[R69] Thibault N, Anderskouv K, Bjerager M, Boldreel LO, Jelby ME, Stemmerik L, Surlyk F (2015). Upper Campanian-Maastrichtian chronostratigraphy of the Skælskør-1 core, Denmark: correlation at the basinal and global scale and implications for changes in seasurface temperatures. Lethaia.

[R70] Thibault N, Harlou R, Schovsbo N, Schiøler P, Minoletti F, Galbrun B, Lauridsen BW, Sheldon E, Stemmerik L, Surlyk F (2012). Upper Campanian-Maastrichtian nannofossil biostratigraphy and high-resolution carbon-isotope stratigraphy of the Danish Basin: Towards a standard δ13C curve for the Boreal Realm. Cretaceous Research.

[R71] Thibault N, Harlou R, Schovsbo NH, Stemmerik L, Surlyk F (2016). Late Cretaceous (late Campanian-Maastrichtian) sea-surface temperature record of the Boreal Chalk Sea. Climate of the Past.

[R72] Vellekoop J, Kaskes P, Sinnesael M, Huygh J, Déhais T, Jagt JWM, Speijer RP, Claeys P (2022). A new age model and chemostratigraphic framework for the Maastrichtian type area (southeastern Netherlands, northeastern Belgium). Newsletters on Stratigraphy.

[R73] Voigt S, Gale AS, Jung C, Jenkyns HC (2012). Global correlation of Upper Campanian − Maastrichtian successions using carbon-isotope stratigraphy: development of a new Maastrichtian timescale. Newsletters on Stratigraphy.

[R74] Voigt S, Schönfeld J (2010). Cyclostratigraphy of the reference section for the Cretaceous white chalk of northern Germany, Lägerdorf-Kronsmoor: A late Campanian-early Maastrichtian orbital time scale. Palaeogeography, Palaeoclimatology, Palaeoecology.

[R75] Vonhof HB, Jagt JWM, Immenhauser A, Smit J, Van den Berg Y, Saher M, Keutgen N, Reijmer JJG, Jagt JWM, Jagt-Yazykova EA, Schins WJH (2011). Belemnite-based strontium carbon and oxygen isotope stratigraphy of the Maastrichtian stratotype area. A tribute to the late Felder brothers − pioneers of Limburg geology and prehistoric archaeology. Netherlands Journal of Geosciences / Geologie en Mijnbouw.

[R76] Von Schlotheim EF, Leonhard CC (1813). Beiträge zur Naturgeschichte der Versteinerungen in geognostischer Hinsicht. Leonhard’s Taschenbuch für die gesamte Mineralogie mit Hinsicht auf die neuesten Entdeckungen.

[R77] Whitfield RP (1892). Gastropoda and Cephalopoda of the Raritan Clays and Greensand Marls of New Jersey. United States Geological Survey, Monograph.

[R78] Wiese F, Košták M, Wood CJ (2009). The Upper Cretaceous belemnite *Praeactinocamax plenus* (Blainville, 1827) from Lower Saxony (Upper Cenomanian, northwest Germany) and its distribution pattern in Europe. Paläontologische Zeitschrift.

[R79] Wilmsen M, Engelke J, Linnert C, Mutterlose J, Niebuhr B (2019). A Boreal reference section revisited (Kronsmoor, northern Germany): high-resolution stratigraphic calibration of the Campanian-Maastrichtian boundary interval (Upper Cretaceous). Newsletters on Stratigraphy.

[R80] Wilmsen M, Niebuhr B (2017). High-resolution Campanian-Maastrichtian carbon and oxygen stable isotopes of bulk-rock and skeletal components: palaeoceanographic and palaeoenvironmental implications for the Boreal shelf sea. Acta Geologica Polonica.

[R81] Zakharov YD, Melnikov ME, Popov AM, Pletnev SP, Khudik VD, Punina TA (2012). Cephalopod and brachiopod fossils from the Pacific: Evidence from the Upper Cretaceous of the Magellan Seamounts. Geobios.

[R82] Zakharov YD, Pletnev SP, Melnikov ME, Smyshlyaeva OP, Khudik VD, Evseev GA, Punina TA, Popov AM (2007). The first finds of Cretaceous belemnites from the Magellan Rise, Pacific Ocean. Russian Journal of Pacific Geology.

[R83] Zakharov YD, Tanabe K, Safronov PP, Smyshlyaeva OP (2014). New data on microstructure and isotopic composition of some cephalopods from the Upper Cretaceous of North America and Europe: significance for oxygen isotope palaeotemperature measurements. Denisia.

